# Notice of duplicate publication: Differences in medical services in Nordic general practice: a comparative survey from the QUALICOPC study

**DOI:** 10.1080/02813432.2023.2228052

**Published:** 2023-07-15

Bjerve Eide, T., Straand, J., Björklund, C., Kosunen, E., Thorgeirsson, O., Vedsted, P. and Rosvold, E.O. Differences in medical services in Nordic general practice: a comparative survey from the QUALICOPC study. *Scandinavian Journal of Primary Health Care*. 2017: https://doi.org/10.1080/02813432.2017.1358856

Please note that this article has been removed from *Scandinavian Journal of Primary Health Care* as it is a Duplicate Publication of:

Bjerve Eide, T., Straand, J., Björklund, C., Kosunen, E., Thorgeirsson, O., Vedsted, P. and Rosvold, E.O. Differences in medical services in Nordic general practice: a comparative survey from the QUALICOPC study. *Scandinavian Journal of Primary Health Care*. 2017;35(2):153–161. https://doi.org/10.1080/02813432.2017.1333323

